# Early Life Stress- and Drug-Induced Histone Modifications Within the Ventral Tegmental Area

**DOI:** 10.3389/fcell.2020.588476

**Published:** 2020-09-30

**Authors:** Ryan D. Shepard, Fereshteh S. Nugent

**Affiliations:** Department of Pharmacology, Edward Hebert School of Medicine, Uniformed Services University of the Health Sciences, Bethesda, MD, United States

**Keywords:** ventral tegmental area, VTA, epigenetic, histone, acetylation, methylation, dopaminylation

## Abstract

Psychiatric illnesses are a major public health concern due to their prevalence and heterogeneity of symptom presentation resulting from a lack of efficacious treatments. Although dysregulated dopamine (DA) signaling has been observed in a myriad of psychiatric conditions, different pathophysiological mechanisms have been implicated which impede the development of adequate treatments that work across all patient populations. The ventral tegmental area (VTA), a major source of DA neurons in the brain reward pathway, has been shown to have altered activity that contributes to reward dysregulation in mental illnesses and drug addiction. It has now become better appreciated that epigenetic mechanisms contribute to VTA DA dysfunction, such as through histone modifications, which dynamically regulate transcription rates of critical genes important in synaptic plasticity underlying learning and memory. Here, we provide a focused review on differential histone modifications within the VTA observed in both humans and animal models, as well as their relevance to disease-based phenotypes, specifically focusing on epigenetic dysregulation of histones in the VTA associated with early life stress (ELS) and drugs of abuse. Locus- and cell-type-specific targeting of individual histone modifications at specific genes within the VTA presents novel therapeutic targets which can result in greater efficacy and better long-term health outcomes in susceptible individuals that are at increased risk for substance use and psychiatric disorders.

## Introduction

Psychiatric disorders pose an extraordinary challenge to healthcare professionals due to their high prevalence and distribution globally. This places an extreme burden on healthcare systems from both an economic and resource standpoint due to a lack of sufficient understanding of the development and progression of various psychiatric disorders. While various degrees of genetic and phenotypic heterogeneity exist among patients, exposure to environmental risk factors contributes to individual variability through their effects on developmental organization of functional connections within discrete brain networks. This creates challenges in the development of therapeutics that are efficacious, long lasting, and generalizable across patient populations.

**FIGURE 1:**
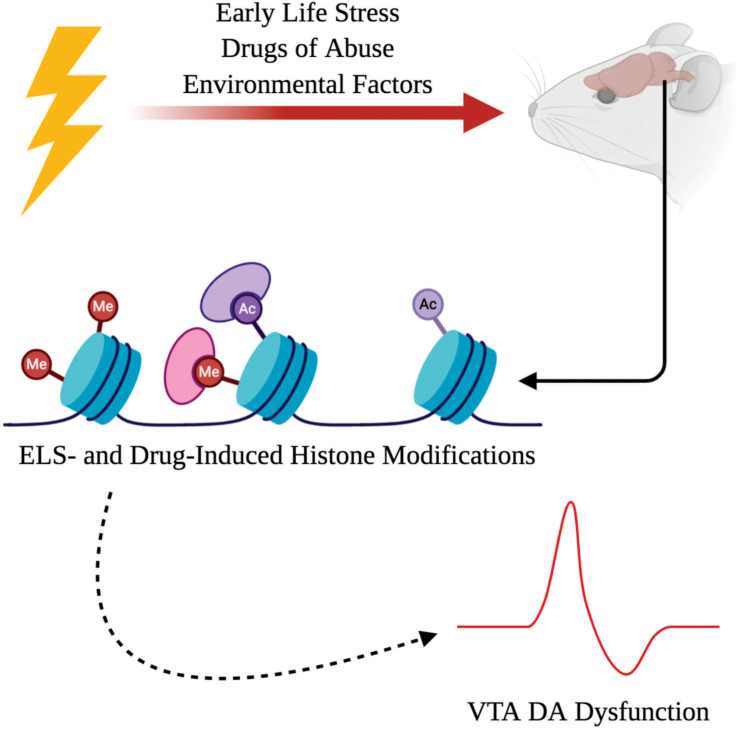
Adverse interactions with the environment, such as early life stress (ELS) or exposure to drugs of abuse, can induce chromatin remodeling as a result of histone modifications. ELS- and drug-induced histone modifications can drive dopamine (DA) dysfunction within the ventral tegmental area (VTA) which contributes to the psychopathology observed in psychiatric illnesses.

It has become more apparent that environmental interactions can impact both the development and function of the central nervous system (CNS). Although some psychiatric diseases have been found to have an underlying genetic basis due to mutations in the coding of the DNA itself, there has been increasing interest in the role of disease-based alterations to the epigenome (Klengel and Binder, 2015). Coined by Waddington, “epigenetic” changes refer to genomic modifications that do not alter the coding of DNA within an organism, but rather influence chromatin architecture which regulates the rate of gene transcription (Berger et al., 2009; Deans and Maggert, 2015). There are various changes to the epigenome that can either enhance or repress transcriptional rates such as histone modifications (Kouzarides, 2007), DNA methylation (Greenberg and Bourc’his, 2019), and non-coding RNAs (Wei et al., 2017). This focused review centers on the role of histone modifications within the ventral tegmental area (VTA)—one of the major sources of dopamine (DA) neurons in the mesolimbic reward pathway; however, we acknowledge there is an extensive role for other epigenetic mechanisms such as DNA methylation and non-coding RNAs in psychiatric disorders (Kuehner et al., 2019). Ultimately, this review will provide a succinct and up-to-date summary of major findings on different histone modifications observed in the VTA specifically following exposure to ELS and drugs of abuse as environmental risks for psychiatric disorders (Figure 1).

### VTA Dysfunction in Psychiatric Illnesses

The VTA is a heterogeneous structure consisting of DA (Zhang et al., 2010), GABAergic (Margolis et al., 2012), and glutamatergic neurons (Yamaguchi et al., 2007; Hnasko et al., 2012) with DA neurons representing the greatest percentage in total cellular composition (Morales and Margolis, 2017). The VTA has been traditionally studied within the context of reward- and motivated learning in that exposure to naturally rewarding stimuli results in DA release which encodes for reward prediction errors that reinforce reward-related behaviors (D’Ardenne et al., 2008; Schultz, 2010; Cohen et al., 2012; Steinberg et al., 2014; Keiflin and Janak, 2015). However, in juxtaposition, VTA DA neurons also regulate aversion and incentive salience (Tan et al., 2012; Lammel et al., 2014; Root et al., 2018). The VTA receives various excitatory inputs from structures such as the medial prefrontal cortex (mPFC), lateral dorsal tegmental area (LDTg), lateral hypothalamus (LH), bed nucleus of stria terminalis (BNST), and the lateral habenula (LHb) (Au-Young et al., 1999; Tzschentke and Schmidt, 2000; Georges and Aston-Jones, 2001; Omelchenko and Sesack, 2005; Caille et al., 2009; Lammel et al., 2012; Kempadoo et al., 2013; Brown and Shepard, 2016). Additionally, VTA DA neurons receive inhibitory signals from GABAergic neurons within the VTA (Margolis et al., 2012), as well as from the rostromedial tegmental nucleus (Kaufling et al., 2009) (RMTg; sometimes referred to as the “tail of the VTA”) and nucleus accumbens (NAc) (Matsui et al., 2014; Edwards et al., 2017). Stimulation of these GABAergic populations restrain the release of DA from the VTA (Matsui and Williams, 2011; Poller et al., 2011; van Zessen et al., 2012; Matsui et al., 2014), but these GABAergic structures can also indirectly mediate the inhibitory effects on DA signaling from glutamatergic projections, such as the LHb (Ji and Shepard, 2007; Matsumoto and Hikosaka, 2007; Omelchenko et al., 2009; Brown et al., 2017). Of interest, VTA GABAergic interneurons also receive direct GABAergic inputs from the NAc which promote DA disinhibition (Xia et al., 2011) and therefore can contribute to reward dysregulation independent of DA signaling (Brown et al., 2012; Creed et al., 2014). VTA glutamatergic and GABAergic synapses exhibit both drug-induced and stress-induced plasticity (Nugent et al., 2007; Niehaus et al., 2010; Bellone and Luscher, 2012; Polter and Kauer, 2014; Authement et al., 2015; Langlois and Nugent, 2017; Polter et al., 2018), demonstrating how synaptic dysfunction at distinct, yet interconnected neural circuits, can dysregulate VTA DA neuronal responses and promote the formation of more habitual and compulsive stress-/drug-related behaviors. Importantly, stress and drugs of abuse “hijack” the brain reward circuitry and alter DA signaling from the VTA (Volkow and Morales, 2015; Langlois and Nugent, 2017; Bellone et al., 2020; Doyle and Mazei-Robison, 2020). One of the hallmarks of depression is anhedonia (inability to perceive pleasure) which can also result from aberrant VTA DA signaling that mediates reward deficits (Heshmati and Russo, 2015; Belujon and Grace, 2017). Human imaging studies have proven useful in helping to corroborate the role of DA dysregulation in psychiatric illnesses and substance use disorders (SUDs) (Volkow et al., 2007; Koob and Volkow, 2010; Shen et al., 2012). Altogether preclinical and clinical data highlight that VTA DA dysfunction is a major contributor in the pathophysiology of reward dysregulation and psychiatric disorders.

### Histone Modifications

Histone modifications dynamically regulate the chromatin structure that influences the rate at which genes are accessed for transcription and subsequent translation. Transcriptional regulation has been widely demonstrated as an important set of processes that can both temporally and spatially define expression patterns of critical proteins and substrates which underlie important processes such as synaptic plasticity and neuronal excitability (Jiang et al., 2008; Sultan and Day, 2011). Specifically, alterations to chromatin structure are impacted by chromatin association with histones, which are octomeric proteins consisting of a combination of four different protein subunits: H2A, H2B, H3, and H4 (Luger et al., 1997); together, these form the nucleosome. These protein subunits have amino acid residues (also referred to as histone tails) that can be chemically modified by various enzymes (Allfrey and Mirsky, 1964; Allfrey et al., 1964). Chemical modifications to these amino acid residues affect how tightly associated DNA chromatin is bound to the histone. Thus, the chromatin state can either be more tightly associated or more relaxed with respect to the physical distance from the histone. A multitude of histone modifications have been documented including: methylation (Greer and Shi, 2012), acetylation (Graff and Tsai, 2013), phosphorylation (Rossetto et al., 2012), palmitylation (Wilson et al., 2011), polyADP-ribosylation (Martinez-Zamudio and Ha, 2012), sumoylation (Shiio and Eisenman, 2003), and ubiquitination (Zhang, 2003). Most recently, serotonylation (Farrelly et al., 2019; Fu and Zhang, 2019; Zlotorynski, 2019) and dopaminylation (Lepack et al., 2020) are two new types of histone modifications that have been observed. The importance of temporal regulation of the chromatin structure via histone modifications cannot be underestimated due to its importance in human development and the possible contribution of “histone code” alteration in disease (Borrelli et al., 2008).

### Acetylation

Histone acetylation is the most understood histone modification in the CNS (Maze et al., 2013) and is dynamically regulated by two different classes of enzymes: histone acetyltransferases (HATs) and histone deacetylases (HDACs). HATs transfer acetyl groups to histone tails which results in chromatin relaxation, generally increasing transcription rates; HDACs remove acetyl groups which increase the chromatin-histone interaction and thus decrease the rate of transcription (Marmorstein and Zhou, 2014). The role of HATs in the VTA have not been extensively studied; however, one interesting study highlighted that cocaine abstinence promoted an increase in BDNF transcripts due to histone acetylation by CREB binding protein (CBP) in the VTA (Schmidt et al., 2012). Given this observation, further investigation into the contribution of HATs in psychiatric illnesses is warranted.

In contrast, HDACs have received the most attention due to their role in learning and memory (Graff et al., 2014; Mahgoub and Monteggia, 2014). Briefly, histone deacetylases can be classified based on the co-factor required for their activation, being either zinc-dependent or NAD-dependent (Haberland et al., 2009). The zinc-dependent HDACs consist of class I (HDAC1, 2, 3, and 8), class II (HDAC4, 5, 6, 7, 9, and 10), and class IV (HDAC11). NAD-dependent HDACs are also referred to as sirtuins (SIRT1, 2, 3, 4, 5, 6, 7, 8). Importantly, class I, II, and IV HDACs are primarily neuronal specific with class I HDACs residing in the nucleus and class II HDACs shuttling between nucleus and cytoplasm (Broide et al., 2007).

Several studies from our lab and other groups have corroborated the role of HDAC-mediated alterations to VTA histone acetylation that contributes to VTA DA dysfunction. Using a rat model of ELS (maternal deprivation, MD), we have found that MD induces GABAergic metaplasticity at GABAergic synapses onto VTA DA neurons that preferentially promotes long-term depression (LTD) (Authement et al., 2015). Moreover, MD-induced GABAergic synaptic dysfunction in VTA DA neurons was associated with altered A-kinase anchoring protein (AKAP150) signaling and decreased BDNF abundance possibly through HDAC2-mediated histone modification (Shepard et al., 2018). Specifically, HDAC2 was upregulated in VTA DA neurons with concomitant decreases in acetylation at H3K9 in protein isolates harvested from VTA tissues. Additionally, MD also increased VTA DA neuronal excitability involving altered AKAP150 signaling (Shepard et al., 2020). Interestingly, administration of CI-994 (a selective class I histone deacetylase inhibitor, HDACi) recovered GABAergic plasticity, histone acetylation, BDNF abundance, (Authement et al., 2015; Shepard et al., 2018), and VTA DA excitability (Shepard et al., 2020). Indeed, transcriptional regulation of the *Bdnf* gene through histone modifications including deacetylation of H3K9 via HDAC2 has also been reported (Chen et al., 2003; Wang et al., 2014; Chen and Chen, 2017). This raises the possibility that MD may lead to transcriptional repression of *Bdnf* gene in VTA DA neurons through increased HDAC2 occupancy at *Bdnf* promoters and histone H3K9 deacetylation in specific *Bdnf* gene promoters. MD-induced dysregulation of BDNF signaling in the VTA could potentially impact synaptic plasticity and VTA DA excitability through its effects on AKAP150 complex. In fact, BDNF signaling has previously been shown to regulate proteasome-dependent synapse remodeling and synaptic protein concentrations including the scaffold protein AKAP150 (Jia et al., 2008). Recent evidence also suggests that the reduction of AKAP150 from the postsynaptic density (PSD) via proteasomal degradation results in endocytosis of GluA1-containing AMPA receptors and promotion of LTD in mouse-cultured neurons (Cheng et al., 2020). Therefore, it will be worthwhile to investigate whether MD-induced epigenetic dysregulation of BDNF expression through HDAC2-histone hypoacetylation promotes degradation of AKAP150 at GABAergic synapses. This can act in concert with MD-induced disruption of AKAP150-PKA anchoring at GABAergic synapses (Shepard et al., 2018) to increase endocytosis of GABA_A_ receptors and induce AKAP150-dependent LTD in VTA DA neurons (Dacher et al., 2013; Authement et al., 2015).

Drugs of abuse have also been shown to induce changes in histone acetylation (Renthal and Nestler, 2009), which possibly contribute to the etiology and maintenance of SUDs. Studies involving alcohol administration have also showed that alcohol increases HDAC2 expression, decreases acetylation of H3K9 and dysregulates GABA_A_R signaling in the VTA; the effects of alcohol can be ameliorated with HDAC inhibition (Arora et al., 2013; You et al., 2018). In fact, it has been documented that the metabolic breakdown of ethanol and production of acetate can contribute to changes in histone acetylation (Mews et al., 2019). Given that HDAC inhibition could attenuate alcohol-seeking behaviors, this further builds on evidence that dysregulated histone acetylation dynamics can contribute to maladaptive behaviors (Jeanblanc et al., 2015; Simon-O’Brien et al., 2015); however, whether these observations are exclusive to the VTA or involve other brain structures have not been fully elucidated. Morphine and its precursor, heroin, also have been observed to alter histone acetylation within the VTA (Authement et al., 2016; Xu et al., 2016). Moreover, acute exposure to morphine has been shown to dysregulate VTA synaptic transmission. Interestingly, HDACi treatment can normalize synaptic transmission and histone hypoacetylation in the VTA that are associated with acute morphine administration (Authement et al., 2016). Collectively, these studies highlight that HDAC-mediated histone modifications (such as histone hypoacetylation) within the VTA can contribute to transcriptional changes in BDNF signaling, AKAP150-dependent synaptic remodeling and stress- and drug-induced synaptic plasticity in VTA neurons that are reversible. Given that targeting HDACs with HDACi can ameliorate VTA synaptic dysfunction and the associated stress- and drug-related behaviors (Covington et al., 2009; Machado-Vieira et al., 2011), the use of HDACi could prove useful in treating or preventing neuropsychiatric disorders that stem from VTA dysfunction.

### Methylation

Similar to histone acetylation, histone methylation is regulated by two classes of enzymes: histone methyltransferases (HMTs) and histone demethylases (HDMs). However, unlike histone acetylation, the addition and/or removal of methyl groups from histone tails by HMTs or HDMs, respectively, is not limited to a single chemical alteration. In fact, histone tails can either be mono-, di-, or tri-methylated depending on the amino acid residue which can have differential effects on the rate of transcription based on the methylation site (Black et al., 2012). Methylation of histones is accomplished by three families of HMTs that use S-adenosylmethionine (SAM) as the substrate that transfers the methyl group(s): (Su(var)3-9, enhancer of Zeste, trithorax)-domain containing proteins (Rea et al., 2000), DOT1-like proteins (Nguyen and Zhang, 2011), and protein arginine N-methyltransferases (PRMTs) (Di Lorenzo and Bedford, 2011). Demethylation of histones is carried out by two classes: amine oxidase LSD1 (KDM1) (Shi et al., 2004) and the Jumonji (JmjC) domain protein family (Klose et al., 2006; Tsukada et al., 2006). Given that histone acetylation can regulate VTA transcriptional activity and subsequently physiology, it is not surprising that alterations to histone methylation dynamics can also contribute to VTA pathophysiology.

In one study, decreased levels of BDNF transcript from VTA tissues were observed in samples obtained from both postmortem heroin users and rodent models (Koo et al., 2015). Chronic morphine was shown to increase trimethylation at both histone-3-lysine-4 (H3K4me3) and histone-3-lysine-27 (H3K27me3) which was associated with the BDNF II promoter. It was identified that H3K27me3 was mediated by the HMT enhancer of zeste homolog 2 (EZH2) in that overexpression and knockdown resulted in a decrease and increase of BDNF, respectively. Additionally, the actions of EZH2 on histone methylation impacted morphine’s behavioral effects. However, only one study, the findings of this study, strongly suggests that overactivity of HMTs might be involved with drugs of abuse, such as morphine. Given that less is known about histone methylation, more research is required to understand if these alterations are specific to morphine or if they can extrapolate to other conditions.

### Dopaminylation

Most recently, novel modifications to histones have been identified that can be modulated by neurotransmitters themselves: specifically, serotonylation (Farrelly et al., 2019; Fu and Zhang, 2019; Zlotorynski, 2019) and dopaminylation (Lepack et al., 2020). These intriguing findings suggest how dysregulated neurotransmission of monoamines, such as serotonin (5-HT) or DA themselves, can impact the epigenome to produce neuroadaptations involved in psychiatric diseases. Postmortem analysis of VTA tissue from cocaine users identified decreased dopaminylation of H3Q5 which was also observed in a rodent model of cocaine administration immediately after withdrawal; however, the levels of H3Q5 dopaminylation increased during prolonged withdrawal (30 days) (Lepack et al., 2020). This dopaminylation also was associated with altered transcription and increased release of DA into the NAc. Taken together, this novel finding opens up a previously unknown mechanism involved in histone regulation and suggests that monoamines themselves alters transcription of genes with subsequent changes in VTA physiology and related behaviors.

## Conclusion

Precision-based medicine and advancements in genetic sequencing technologies have rapidly changed how we understand disease states, including psychiatric disorders. It has now become more greatly accepted that there is a genetic basis to many neuropsychiatric conditions. More importantly, the role of environmental modulation of epigenetic processes is being established through human and animal studies. This also poses an important and interesting question as to whether epigenetic alterations can be inherited (Heard and Martienssen, 2014). Transgenerational epigenetic inheritance has been documented in the case of DNA methylation (Sen et al., 2015), but not yet in the case of histone modifications. This observation begs the question as to whether epigenetic modifications need to be targeted during a critical period or can be possibly used pre-emptively to prevent adaptations that will result in psychiatric illnesses later in life. Thus, targeting enzymes mediating histone modifications should be further investigated and represent new pharmacological targets. In fact, certain compounds such as HDACi are already used in the treatment of cancer (West and Johnstone, 2014) and are being considered for use in psychiatric conditions (Covington et al., 2009; Machado-Vieira et al., 2011). One of the most interesting recent discoveries has been the possible contribution of serotonylation (Farrelly et al., 2019; Fu and Zhang, 2019; Zlotorynski, 2019) and dopaminylation (Lepack et al., 2020) to histone modifications—two previously unknown epigenetic mechanisms. These two new mechanisms of histone modification alone add a new layer of complexity when studying epigenetic contributions to psychiatric disorders, but also give exciting and new targets for the development of therapies.

Moreover, one of the largest challenges in assessing epigenetic alterations, such as histone modifications, is understanding how they affect the CNS with region- and cell-type and locus-specificity. Previous experimental approaches of either transcriptional activation or suppression make it difficult to dissect contributions of epigenetic alterations to physiology and behavior due to artificial changes in gene expression that are not physiological (Yim et al., 2020). The advent of new epigenome-editing tools offer opportunities for precise locus-specific post-translational histone modifications including histone acetylation and histone methylation at specific genes in a single cell type/brain region (Hamilton et al., 2018b; Xu and Heller, 2019; Yim et al., 2020). The emerging and exciting field of neuroepigenetic editing tools allows for interrogation of epigenetic modifications in discreet brain regions and provides the opportunity to study causal relationships between gene transcriptional activity and the subsequent neural plasticity and behavior. For example, drug-induced activation of *Fosb* gene through increased histone acetylation (Levine et al., 2005) and decreased histone methylation (Maze et al., 2010) at its promoter has been observed, although the functional relevance of these correlated global histone post-translational modifications to drug-related behavior has been limited. To overcome this limitation, locus-specific targeting of the *Fosb* promoter with engineered transcription factors fused to zinc-finger proteins (ZFPs) facilitated bidirectional regulation of ΔFosB expression in mouse NAc neurons. Upregulation and downregulation was achieved using the transcriptional activator, p65, which promoted histone acetylation at H3K9/14 and the transcriptional repressor, G9a (promoting histone methylation at H3 lysine 9 dimethylation, H3K9me2) specifically at *Fosb*, respectively. Additionally, this bidirectional regulation of *Fosb* gene expression through these locus-specific histone manipulations was sufficient to induce opposing cocaine-related behaviors. Furthermore, they provided a direct causal link between H3K9me2-mediated reduction in *Fosb/ΔFosb* expression in mouse NAc and the promotion of depression- and anxiety-like behaviors after social stress (Heller et al., 2014). Similarly, in another study, the same viral-epigenetic approach was employed to induce these complementary histone modifications at the cyclin-dependent kinase 5 (Cdk5) locus in mouse NAc and found that Cdk5-targeted H3K9/14 acetylation promoted Cdk5 gene expression and cocaine-induced locomotor behavior and resilience to social stress while Cdk5-targeted H3K9me2 induced Cdk5 gene repression and suppressed cocaine-related behaviors (Heller et al., 2016). Cre-dependent cell type-specific expression of *Fosb*-ZFPs in NAc D1- versus D2-medium spiny neurons (MSNs) allowed for cell-specific interrogations and provided compelling evidence for the opposite roles of *Fosb*-targeted histone acetylation and methylation in D1 and D2 MSNs in social defeat stress behavior (Hamilton et al., 2018a). This study also further highlights the necessity to consider differences in cell type-specific alterations to epigenetic modifications and their relationship with neurophysiology and behavior.

Given the robust regulatory role of HDAC-mediated histone modifications in VTA DA function, future epigenetic research should also employ these invaluable *in vivo* neuroepigenetic editing approaches for studying the causal relationships between an epigenetic modification at a single locus induced by stress or drugs of abuse within the specific neuronal populations of the VTA to its downstream functional outcomes at the transcriptional, cellular, circuit and behavioral levels. This locus- and cell type-specific targeted epigenome mapping and manipulation could help create more specific interventions that have more reliable, long-lasting, and efficacious treatment options for patients with psychiatric disease.

## Author Contributions

RS and FN wrote the manuscript. Both authors contributed to the article and approved the submitted version.

## Conflict of Interest

The authors declare that the research was conducted in the absence of any commercial or financial relationships that could be construed as a potential conflict of interest.

## References

[B1] Allfrey V. G. Faulkner R. Mirsky A. E. (1964). Acetylation and methylation of histones and their possible role in the regulation of Rna synthesis. *Proc. Natl. Acad. Sci. U.S.A.* 51 786–794. 10.1073/pnas.51.5.786 14172992 PMC300163

[B2] Allfrey V. G. Mirsky A. E. (1964). Structural modifications of histones and their possible role in the regulation of RNA synthesis. *Science* 144:559. 10.1126/science.144.3618.559 17836360

[B3] Arora D. S. Nimitvilai S. Teppen T. L. McElvain M. A. Sakharkar A. J. You C. (2013). Hyposensitivity to gamma-aminobutyric acid in the ventral tegmental area during alcohol withdrawal: reversal by histone deacetylase inhibitors. *Neuropsychopharmacology* 38 1674–1684. 10.1038/npp.2013.65 23474591 PMC3717553

[B4] Authement M. E. Kodangattil J. N. Gouty S. Rusnak M. Symes A. J. Cox B. M. (2015). Histone deacetylase inhibition rescues maternal deprivation-induced GABAergic metaplasticity through restoration of AKAP signaling. *Neuron* 86 1240–1252. 10.1016/j.neuron.2015.05.024 26050042

[B5] Authement M. E. Langlois L. D. Kassis H. Gouty S. Dacher M. Shepard R. D. (2016). Morphine-induced synaptic plasticity in the VTA is reversed by HDAC inhibition. *J. Neurophysiol.* 116 1093–1103. 10.1152/jn.00238.2016 27306674 PMC5013166

[B6] Au-Young S. M. Shen H. Yang C. R. (1999). Medial prefrontal cortical output neurons to the ventral tegmental area (VTA) and their responses to burst-patterned stimulation of the VTA: neuroanatomical and *in vivo* electrophysiological analyses. *Synapse* 34 245–255. 10529719 10.1002/(SICI)1098-2396(19991215)34:4<245::AID-SYN1>3.0.CO;2-D

[B7] Bellone C. Loureiro M. Luscher C. (2020). Drug-evoked synaptic plasticity of excitatory transmission in the ventral tegmental area. *Cold Spring Harb. Perspect. Med.* 10.1101/cshperspect.a039701 32341062 PMC8015696

[B8] Bellone C. Luscher C. (2012). Drug-evoked plasticity: Do addictive drugs reopen a critical period of postnatal synaptic development? *Front. Mol. Neurosci.* 5:75. 10.3389/fnmol.2012.00075 22715323 PMC3375625

[B9] Belujon P. Grace A. A. (2017). Dopamine system dysregulation in major depressive disorders. *Int. J. Neuropsychopharmacol.* 20 1036–1046. 10.1093/ijnp/pyx056 29106542 PMC5716179

[B10] Berger S. L. Kouzarides T. Shiekhattar R. Shilatifard A. (2009). An operational definition of epigenetics. *Genes Dev.* 23 781–783. 10.1101/gad.1787609 19339683 PMC3959995

[B11] Black J. C. Van Rechem C. Whetstine J. R. (2012). Histone lysine methylation dynamics: establishment, regulation, and biological impact. *Mol. Cell* 48 491–507. 10.1016/j.molcel.2012.11.006 23200123 PMC3861058

[B12] Borrelli E. Nestler E. J. Allis C. D. Sassone-Corsi P. (2008). Decoding the epigenetic language of neuronal plasticity. *Neuron* 60 961–974. 10.1016/j.neuron.2008.10.012 19109904 PMC2737473

[B13] Broide R. S. Redwine J. M. Aftahi N. Young W. Bloom F. E. Winrow C. J. (2007). Distribution of histone deacetylases 1-11 in the rat brain. *J. Mol. Neurosci.* 31 47–58. 10.1007/bf02686117 17416969

[B14] Brown M. T. Tan K. R. O’Connor E. C. Nikonenko I. Muller D. Luscher C. (2012). Ventral tegmental area GABA projections pause accumbal cholinergic interneurons to enhance associative learning. *Nature* 492 452–456. 10.1038/nature11657 23178810

[B15] Brown P. L. Palacorolla H. Brady D. Riegger K. Elmer G. I. Shepard P. D. (2017). Habenula-induced inhibition of midbrain dopamine neurons is diminished by lesions of the rostromedial tegmental nucleus. *J. Neurosci.* 37 217–225. 10.1523/JNEUROSCI.1353-16.2016 28053043 PMC5214632

[B16] Brown P. L. Shepard P. D. (2016). Functional evidence for a direct excitatory projection from the lateral habenula to the ventral tegmental area in the rat. *J. Neurophysiol.* 116 1161–1174. 10.1152/jn.00305.2016 27358317 PMC5013172

[B17] Caille S. Guillem K. Cador M. Manzoni O. Georges F. (2009). Voluntary nicotine consumption triggers *in vivo* potentiation of cortical excitatory drives to midbrain dopaminergic neurons. *J. Neurosci.* 29 10410–10415. 10.1523/JNEUROSCI.2950-09.2009 19692616 PMC6665781

[B18] Chen K. W. Chen L. (2017). Epigenetic regulation of BDNF gene during development and diseases. *Int. J. Mol. Sci.* 18:571. 10.3390/ijms18030571 28272318 PMC5372587

[B19] Chen W. G. Chang Q. Lin Y. Meissner A. West A. E. Griffith E. C. (2003). Derepression of BDNF transcription involves calcium-dependent phosphorylation of MeCP2. *Science* 302 885–889. 10.1126/science.1086446 14593183

[B20] Cheng W. Siedlecki-Wullich D. Catala-Solsona J. Fabregas C. Fado R. Casals N. (2020). Proteasomal-mediated degradation of AKAP150 accompanies AMPAR endocytosis during cLTD. *eNeuro* 7:ENEURO.0218-19.2020. 10.1523/ENEURO.0218-19.2020 32205379 PMC7163082

[B21] Cohen J. Y. Haesler S. Vong L. Lowell B. B. Uchida N. (2012). Neuron-type-specific signals for reward and punishment in the ventral tegmental area. *Nature* 482 85–88. 10.1038/nature10754 22258508 PMC3271183

[B22] Covington H. E. III Maze I. LaPlant Q. C. Vialou V. F. Ohnishi Y. N. Berton O. (2009). Antidepressant actions of histone deacetylase inhibitors. *J. Neurosci.* 29 11451–11460. 10.1523/JNEUROSCI.1758-09.2009 19759294 PMC2775805

[B23] Creed M. C. Ntamati N. R. Tan K. R. (2014). VTA GABA neurons modulate specific learning behaviors through the control of dopamine and cholinergic systems. *Front. Behav. Neurosci.* 8:8. 10.3389/fnbeh.2014.00008 24478655 PMC3897868

[B24] Dacher M. Gouty S. Dash S. Cox B. M. Nugent F. S. (2013). A-kinase anchoring protein-calcineurin signaling in long-term depression of GABAergic synapses. *J. Neurosci.* 33 2650–2660. 10.1523/JNEUROSCI.2037-12.2013 23392692 PMC6619159

[B25] D’Ardenne K. McClure S. M. Nystrom L. E. Cohen J. D. (2008). BOLD responses reflecting dopaminergic signals in the human ventral tegmental area. *Science* 319 1264–1267. 10.1126/science.1150605 18309087

[B26] Deans C. Maggert K. A. (2015). What do you mean, “epigenetic”? *Genetics* 199 887–896. 10.1534/genetics.114.173492 25855649 PMC4391566

[B27] Di Lorenzo A. Bedford M. T. (2011). Histone arginine methylation. *FEBS Lett.* 585 2024–2031. 10.1016/j.febslet.2010.11.010 21074527 PMC3409563

[B28] Doyle M. A. Mazei-Robison M. S. (2020). Opioid-induced molecular and cellular plasticity of ventral tegmental area dopamine neurons. *Cold Spring Harb. Perspect. Med.* 10.1101/cshperspect.a039362 [Epub ahead of print]. 31964652 PMC7371531

[B29] Edwards N. J. Tejeda H. A. Pignatelli M. Zhang S. McDevitt R. A. Wu J. (2017). Circuit specificity in the inhibitory architecture of the VTA regulates cocaine-induced behavior. *Nat. Neurosci.* 20 438–448. 10.1038/nn.4482 28114294

[B30] Farrelly L. A. Thompson R. E. Zhao S. Lepack A. E. Lyu Y. Bhanu N. V. (2019). Histone serotonylation is a permissive modification that enhances TFIID binding to H3K4me3. *Nature* 567 535–539. 10.1038/s41586-019-1024-7 30867594 PMC6557285

[B31] Fu L. Zhang L. (2019). Serotonylation: a novel histone H3 marker. *Signal Transduct. Target. Ther.* 4:15. 10.1038/s41392-019-0048-7 31098305 PMC6509131

[B32] Georges F. Aston-Jones G. (2001). Potent regulation of midbrain dopamine neurons by the bed nucleus of the stria terminalis. *J. Neurosci.* 21 RC160. 10.1523/JNEUROSCI.21-16-j0003.2001 PMC6763146 11473131

[B33] Graff J. Joseph N. F. Horn M. E. Samiei A. Meng J. Seo J. (2014). Epigenetic priming of memory updating during reconsolidation to attenuate remote fear memories. *Cell* 156 261–276. 10.1016/j.cell.2013.12.020 24439381 PMC3986862

[B34] Graff J. Tsai L. H. (2013). Histone acetylation: molecular mnemonics on the chromatin. *Nat. Rev. Neurosci.* 14 97–111. 10.1038/nrn3427 23324667

[B35] Greenberg M. V. C. Bourc’his D. (2019). The diverse roles of DNA methylation in mammalian development and disease. *Nat. Rev. Mol. Cell Biol.* 20 590–607. 10.1038/s41580-019-0159-6 31399642

[B36] Greer E. L. Shi Y. (2012). Histone methylation: a dynamic mark in health, disease and inheritance. *Nat. Rev. Genet.* 13 343–357. 10.1038/nrg3173 22473383 PMC4073795

[B37] Haberland M. Montgomery R. L. Olson E. N. (2009). The many roles of histone deacetylases in development and physiology: implications for disease and therapy. *Nat. Rev. Genet.* 10 32–42. 10.1038/nrg2485 19065135 PMC3215088

[B38] Hamilton P. J. Burek D. J. Lombroso S. I. Neve R. L. Robison A. J. Nestler E. J. (2018a). Cell-type-specific epigenetic editing at the fosb gene controls susceptibility to social defeat stress. *Neuropsychopharmacology* 43 272–284. 10.1038/npp.2017.88 28462942 PMC5729576

[B39] Hamilton P. J. Lim C. J. Nestler E. J. Heller E. A. (2018b). Neuroepigenetic editing. *Methods Mol. Biol.* 1767 113–136. 10.1007/978-1-4939-7774-1_5 29524131 PMC6047758

[B40] Heard E. Martienssen R. A. (2014). Transgenerational epigenetic inheritance: myths and mechanisms. *Cell* 157 95–109. 10.1016/j.cell.2014.02.045 24679529 PMC4020004

[B41] Heller E. A. Cates H. M. Pena C. J. Sun H. Shao N. Feng J. (2014). Locus-specific epigenetic remodeling controls addiction- and depression-related behaviors. *Nat. Neurosci.* 17 1720–1727. 10.1038/nn.3871 25347353 PMC4241193

[B42] Heller E. A. Hamilton P. J. Burek D. D. Lombroso S. I. Pena C. J. Neve R. L. (2016). Targeted epigenetic remodeling of the Cdk5 gene in nucleus accumbens regulates cocaine- and stress-evoked behavior. *J. Neurosci.* 36 4690–4697. 10.1523/JNEUROSCI.0013-16.2016 27122028 PMC4846670

[B43] Heshmati M. Russo S. J. (2015). Anhedonia and the brain reward circuitry in depression. *Curr. Behav. Neurosci. Rep.* 2 146–153. 10.1007/s40473-015-0044-3 26525751 PMC4626008

[B44] Hnasko T. S. Hjelmstad G. O. Fields H. L. Edwards R. H. (2012). Ventral tegmental area glutamate neurons: electrophysiological properties and projections. *J. Neurosci.* 32 15076–15085. 10.1523/JNEUROSCI.3128-12.2012 23100428 PMC3685320

[B45] Jeanblanc J. Lemoine S. Jeanblanc V. Alaux-Cantin S. Naassila M. (2015). The class I-Specific HDAC Inhibitor MS-275 decreases motivation to consume alcohol and relapse in heavy drinking rats. *Int. J. Neuropsychopharmacol.* 18:pyv029. 10.1093/ijnp/pyv029 25762717 PMC4576514

[B46] Ji H. Shepard P. D. (2007). Lateral habenula stimulation inhibits rat midbrain dopamine neurons through a GABA(A) receptor-mediated mechanism. *J. Neurosci.* 27 6923–6930. 17596440 10.1523/JNEUROSCI.0958-07.2007 PMC6672239

[B47] Jia J. M. Chen Q. Zhou Y. Miao S. Zheng J. Zhang C. (2008). Brain-derived neurotrophic factor-tropomyosin-related kinase B signaling contributes to activity-dependent changes in synaptic proteins. *J. Biol. Chem.* 283 21242–21250. 10.1074/jbc.M800282200 18474605 PMC3258936

[B48] Jiang Y. Langley B. Lubin F. D. Renthal W. Wood M. A. Yasui D. H. (2008). Epigenetics in the nervous system. *J. Neurosci.* 28 11753–11759. 10.1523/JNEUROSCI.3797-08.2008 19005036 PMC3844836

[B49] Kaufling J. Veinante P. Pawlowski S. A. Freund-Mercier M. J. Barrot M. (2009). Afferents to the GABAergic tail of the ventral tegmental area in the rat. *J. Comp. Neurol.* 513 597–621. 10.1002/cne.21983 19235223

[B50] Keiflin R. Janak P. H. (2015). Dopamine prediction errors in reward learning and addiction: from theory to neural circuitry. *Neuron* 88 247–263. 10.1016/j.neuron.2015.08.037 26494275 PMC4760620

[B51] Kempadoo K. A. Tourino C. Cho S. L. Magnani F. Leinninger G. M. Stuber G. D. (2013). Hypothalamic neurotensin projections promote reward by enhancing glutamate transmission in the VTA. *J. Neurosci.* 33 7618–7626. 10.1523/JNEUROSCI.2588-12.2013 23637156 PMC3865559

[B52] Klengel T. Binder E. B. (2015). Epigenetics of stress-related psychiatric disorders and gene x environment interactions. *Neuron* 86 1343–1357. 10.1016/j.neuron.2015.05.036 26087162

[B53] Klose R. J. Kallin E. M. Zhang Y. (2006). JmjC-domain-containing proteins and histone demethylation. *Nat. Rev. Genet.* 7 715–727. 10.1038/nrg1945 16983801

[B54] Koo J. W. Mazei-Robison M. S. LaPlant Q. Egervari G. Braunscheidel K. M. Adank D. N. (2015). Epigenetic basis of opiate suppression of Bdnf gene expression in the ventral tegmental area. *Nat. Neurosci.* 18 415–422. 10.1038/nn.3932 25643298 PMC4340719

[B55] Koob G. F. Volkow N. D. (2010). Neurocircuitry of addiction. *Neuropsychopharmacology* 35 217–238. 10.1038/npp.2009.110 19710631 PMC2805560

[B56] Kouzarides T. (2007). Chromatin modifications and their function. *Cell* 128 693–705. 10.1016/j.cell.2007.02.005 17320507

[B57] Kuehner J. N. Bruggeman E. C. Wen Z. Yao B. (2019). Epigenetic regulations in neuropsychiatric disorders. *Front. Genet.* 10:268. 10.3389/fgene.2019.00268 31019524 PMC6458251

[B58] Lammel S. Lim B. K. Malenka R. C. (2014). Reward and aversion in a heterogeneous midbrain dopamine system. *Neuropharmacology* 76(Pt B), 351–359. 10.1016/j.neuropharm.2013.03.019 23578393 PMC3778102

[B59] Lammel S. Lim B. K. Ran C. Huang K. W. Betley M. J. Tye K. M. (2012). Input-specific control of reward and aversion in the ventral tegmental area. *Nature* 491 212–217. 10.1038/nature11527 23064228 PMC3493743

[B60] Langlois L. D. Nugent F. S. (2017). Opiates and plasticity in the ventral tegmental area. *ACS Chem. Neurosci.* 8 1830–1838. 10.1021/acschemneuro.7b00281 28768409 PMC5775906

[B61] Lepack A. E. Werner C. T. Stewart A. F. Fulton S. L. Zhong P. Farrelly L. A. (2020). Dopaminylation of histone H3 in ventral tegmental area regulates cocaine seeking. *Science* 368 197–201. 10.1126/science.aaw8806 32273471 PMC7228137

[B62] Levine A. A. Guan Z. Barco A. Xu S. Kandel E. R. Schwartz J. H. (2005). CREB-binding protein controls response to cocaine by acetylating histones at the fosB promoter in the mouse striatum. *Proc. Natl. Acad. Sci. U.S.A.* 102 19186–19191. 10.1073/pnas.0509735102 16380431 PMC1323217

[B63] Luger K. Mader A. W. Richmond R. K. Sargent D. F. Richmond T. J. (1997). Crystal structure of the nucleosome core particle at 2.8 A resolution. *Nature* 389 251–260. 10.1038/38444 9305837

[B64] Machado-Vieira R. Ibrahim L. Zarate C. A. Jr. (2011). Histone deacetylases and mood disorders: epigenetic programming in gene-environment interactions. *CNS Neurosci. Ther.* 17 699–704. 10.1111/j.1755-5949.2010.00203.x 20961400 PMC3026916

[B65] Mahgoub M. Monteggia L. M. (2014). A role for histone deacetylases in the cellular and behavioral mechanisms underlying learning and memory. *Learn. Mem.* 21 564–568. 10.1101/lm.036012.114 25227251 PMC4175496

[B66] Margolis E. B. Toy B. Himmels P. Morales M. Fields H. L. (2012). Identification of rat ventral tegmental area GABAergic neurons. *PLoS One* 7:e42365. 10.1371/journal.pone.0042365 22860119 PMC3409171

[B67] Marmorstein R. Zhou M. M. (2014). Writers and readers of histone acetylation: structure, mechanism, and inhibition. *Cold Spring Harb. Perspect. Biol.* 6:a018762. 10.1101/cshperspect.a018762 24984779 PMC4067988

[B68] Martinez-Zamudio R. Ha H. C. (2012). Histone ADP-ribosylation facilitates gene transcription by directly remodeling nucleosomes. *Mol. Cell Biol.* 32 2490–2502. 10.1128/MCB.06667-11 22547677 PMC3434492

[B69] Matsui A. Jarvie B. C. Robinson B. G. Hentges S. T. Williams J. T. (2014). Separate GABA afferents to dopamine neurons mediate acute action of opioids, development of tolerance, and expression of withdrawal. *Neuron* 82 1346–1356. 10.1016/j.neuron.2014.04.030 24857021 PMC4072256

[B70] Matsui A. Williams J. T. (2011). Opioid-sensitive GABA inputs from rostromedial tegmental nucleus synapse onto midbrain dopamine neurons. *J. Neurosci.* 31 17729–17735. 10.1523/JNEUROSCI.4570-11.2011 22131433 PMC3617570

[B71] Matsumoto M. Hikosaka O. (2007). Lateral habenula as a source of negative reward signals in dopamine neurons. *Nature* 447 1111–1115. 10.1038/nature05860 17522629

[B72] Maze I. Covington H. E. III Dietz D. M. LaPlant Q. Renthal W. Russo S. J. (2010). Essential role of the histone methyltransferase G9a in cocaine-induced plasticity. *Science* 327 213–216. 10.1126/science.1179438 20056891 PMC2820240

[B73] Maze I. Noh K. M. Allis C. D. (2013). Histone regulation in the CNS: basic principles of epigenetic plasticity. *Neuropsychopharmacology* 38 3–22. 10.1038/npp.2012.124 22828751 PMC3521967

[B74] Mews P. Egervari G. Nativio R. Sidoli S. Donahue G. Lombroso S. I. (2019). Alcohol metabolism contributes to brain histone acetylation. *Nature* 574 717–721. 10.1038/s41586-019-1700-7 31645761 PMC6907081

[B75] Morales M. Margolis E. B. (2017). Ventral tegmental area: cellular heterogeneity, connectivity and behaviour. *Nat. Rev. Neurosci.* 18 73–85. 10.1038/nrn.2016.165 28053327

[B76] Nguyen A. T. Zhang Y. (2011). The diverse functions of Dot1 and H3K79 methylation. *Genes Dev.* 25 1345–1358. 10.1101/gad.2057811 21724828 PMC3134078

[B77] Niehaus J. L. Murali M. Kauer J. A. (2010). Drugs of abuse and stress impair LTP at inhibitory synapses in the ventral tegmental area. *Eur. J. Neurosci.* 32 108–117. 10.1111/j.1460-9568.2010.07256.x 20608969 PMC2908505

[B78] Nugent F. S. Penick E. C. Kauer J. A. (2007). Opioids block long-term potentiation of inhibitory synapses. *Nature* 446 1086–1090. 17460674 10.1038/nature05726

[B79] Omelchenko N. Bell R. Sesack S. R. (2009). Lateral habenula projections to dopamine and GABA neurons in the rat ventral tegmental area. *Eur. J. Neurosci.* 30 1239–1250. 10.1111/j.1460-9568.2009.06924.x 19788571 PMC2882626

[B80] Omelchenko N. Sesack S. R. (2005). Laterodorsal tegmental projections to identified cell populations in the rat ventral tegmental area. *J. Comp. Neurol.* 483 217–235. 15678476 10.1002/cne.20417

[B81] Poller W. C. Bernard R. Derst C. Weiss T. Madai V. I. Veh R. W. (2011). Lateral habenular neurons projecting to reward-processing monoaminergic nuclei express hyperpolarization-activated cyclic nucleotid-gated cation channels. *Neuroscience* 193 205–216. 10.1016/j.neuroscience.2011.07.013 21798320

[B82] Polter A. M. Barcomb K. Tsuda A. C. Kauer J. A. (2018). Synaptic function and plasticity in identified inhibitory inputs onto VTA dopamine neurons. *Eur. J. Neurosci.* 47 1208–1218. 10.1111/ejn.13879 29480954 PMC6487867

[B83] Polter A. M. Kauer J. A. (2014). Stress and VTA synapses: implications for addiction and depression. *Eur. J. Neurosci.* 39 1179–1188. 10.1111/ejn.12490 24712997 PMC4019343

[B84] Rea S. Eisenhaber F. O’Carroll D. Strahl B. D. Sun Z. W. Schmid M. (2000). Regulation of chromatin structure by site-specific histone H3 methyltransferases. *Nature* 406 593–599. 10.1038/35020506 10949293

[B85] Renthal W. Nestler E. J. (2009). Histone acetylation in drug addiction. *Semin. Cell Dev. Biol.* 20 387–394. 10.1016/j.semcdb.2009.01.005 19560043 PMC2704458

[B86] Root D. H. Estrin D. J. Morales M. (2018). Aversion or salience signaling by ventral tegmental area glutamate neurons. *iScience* 2 51–62. 10.1016/j.isci.2018.03.008 29888759 PMC5993057

[B87] Rossetto D. Avvakumov N. Cote J. (2012). Histone phosphorylation: a chromatin modification involved in diverse nuclear events. *Epigenetics* 7 1098–1108. 10.4161/epi.21975 22948226 PMC3469451

[B88] Schmidt H. D. Sangrey G. R. Darnell S. B. Schassburger R. L. Cha J. H. Pierce R. C. (2012). Increased brain-derived neurotrophic factor (BDNF) expression in the ventral tegmental area during cocaine abstinence is associated with increased histone acetylation at BDNF exon I-containing promoters. *J. Neurochem.* 120 202–209. 10.1111/j.1471-4159.2011.07571.x 22043863 PMC3243782

[B89] Schultz W. (2010). Dopamine signals for reward value and risk: basic and recent data. *Behav. Brain Funct.* 6:24. 10.1186/1744-9081-6-24 20416052 PMC2876988

[B90] Sen A. Heredia N. Senut M. C. Land S. Hollocher K. Lu X. (2015). Multigenerational epigenetic inheritance in humans: DNA methylation changes associated with maternal exposure to lead can be transmitted to the grandchildren. *Sci. Rep.* 5:14466. 10.1038/srep14466 26417717 PMC4586440

[B91] Shen L. H. Liao M. H. Tseng Y. C. (2012). Recent advances in imaging of dopaminergic neurons for evaluation of neuropsychiatric disorders. *J. Biomed. Biotechnol.* 2012:259349. 10.1155/2012/259349 22570524 PMC3335602

[B92] Shepard R. D. Gouty S. Kassis H. Berenji A. Zhu W. Cox B. M. (2018). Targeting histone deacetylation for recovery of maternal deprivation-induced changes in BDNF and AKAP150 expression in the VTA. *Exp. Neurol.* 309 160–168. 10.1016/j.expneurol.2018.08.002 30102916 PMC6139260

[B93] Shepard R. D. Langlois L. D. Authement M. E. Nugent F. S. (2020). Histone deacetylase inhibition reduces ventral tegmental area dopamine neuronal hyperexcitability involving AKAP150 signaling following maternal deprivation in juvenile male rats. *J. Neurosci. Res.* 98 1457–1467. 10.1002/jnr.24613 32162391 PMC7242123

[B94] Shi Y. Lan F. Matson C. Mulligan P. Whetstine J. R. Cole P. A. (2004). Histone demethylation mediated by the nuclear amine oxidase homolog LSD1. *Cell* 119 941–953. 10.1016/j.cell.2004.12.012 15620353

[B95] Shiio Y. Eisenman R. N. (2003). Histone sumoylation is associated with transcriptional repression. *Proc. Natl. Acad. Sci. U.S.A.* 100 13225–13230. 10.1073/pnas.1735528100 14578449 PMC263760

[B96] Simon-O’Brien E. Alaux-Cantin S. Warnault V. Buttolo R. Naassila M. Vilpoux C. (2015). The histone deacetylase inhibitor sodium butyrate decreases excessive ethanol intake in dependent animals. *Addict. Biol.* 20 676–689. 10.1111/adb.12161 25041570

[B97] Steinberg E. E. Boivin J. R. Saunders B. T. Witten I. B. Deisseroth K. Janak P. H. (2014). Positive reinforcement mediated by midbrain dopamine neurons requires D1 and D2 receptor activation in the nucleus accumbens. *PLoS One* 9:e94771. 10.1371/journal.pone.0094771 24733061 PMC3986242

[B98] Sultan F. A. Day J. J. (2011). Epigenetic mechanisms in memory and synaptic function. *Epigenomics* 3 157–181. 10.2217/epi.11.6 22122279 PMC3350307

[B99] Tan K. R. Yvon C. Turiault M. Mirzabekov J. J. Doehner J. Labouebe G. (2012). GABA Neurons of the VTA drive conditioned place aversion. *Neuron* 73 1173–1183. 10.1016/j.neuron.2012.02.015 22445344 PMC6690362

[B100] Tsukada Y. Fang J. Erdjument-Bromage H. Warren M. E. Borchers C. H. Tempst P. (2006). Histone demethylation by a family of JmjC domain-containing proteins. *Nature* 439 811–816. 10.1038/nature04433 16362057

[B101] Tzschentke T. M. Schmidt W. J. (2000). Functional relationship among medial prefrontal cortex, nucleus accumbens, and ventral tegmental area in locomotion and reward. *Crit. Rev. Neurobiol.* 14 131–142. 11513242

[B102] van Zessen R. Phillips J. L. Budygin E. A. Stuber G. D. (2012). Activation of VTA GABA Neurons Disrupts Reward Consumption. *Neuron* 73 1184–1194. 10.1016/j.neuron.2012.02.016 22445345 PMC3314244

[B103] Volkow N. D. Fowler J. S. Wang G. J. Swanson J. M. Telang F. (2007). Dopamine in drug abuse and addiction: results of imaging studies and treatment implications. *Arch. Neurol.* 64 1575–1579. 10.1001/archneur.64.11.1575 17998440

[B104] Volkow N. D. Morales M. (2015). The brain on drugs: from reward to addiction. *Cell* 162 712–725. 10.1016/j.cell.2015.07.046 26276628

[B105] Wang B. Y. Zhong Y. Zhao Z. Miao Y. (2014). Epigenetic suppression of hippocampal BDNF mediates the memory deficiency induced by amyloid fibrils. *Pharmacol. Biochem. Behav.* 126 83–89. 10.1016/j.pbb.2014.09.009 25242807

[B106] Wei J. W. Huang K. Yang C. Kang C. S. (2017). Non-coding RNAs as regulators in epigenetics (Review). *Oncol. Rep.* 37 3–9. 10.3892/or.2016.5236 27841002

[B107] West A. C. Johnstone R. W. (2014). New and emerging HDAC inhibitors for cancer treatment. *J. Clin. Invest.* 124 30–39. 10.1172/JCI69738 24382387 PMC3871231

[B108] Wilson J. P. Raghavan A. S. Yang Y. Y. Charron G. Hang H. C. (2011). Proteomic analysis of fatty-acylated proteins in mammalian cells with chemical reporters reveals S-acylation of histone H3 variants. *Mol. Cell. Proteomics* 10:M110001198. 10.1074/mcp.M110.001198 21076176 PMC3047146

[B109] Xia Y. Driscoll J. R. Wilbrecht L. Margolis E. B. Fields H. L. Hjelmstad G. O. (2011). Nucleus accumbens medium spiny neurons target non-dopaminergic neurons in the ventral tegmental area. *J. Neurosci.* 31 7811–7816. 10.1523/JNEUROSCI.1504-11.2011 21613494 PMC6633124

[B110] Xu L. Hong Q. Chen X. Xu X. Liu H. Zhou W. (2016). H4K5 histone acetylation of BRG1 is associated with heroin administration rather than addiction. *Exp. Ther. Med.* 12 1929–1933. 10.3892/etm.2016.3517 27588112 PMC4998080

[B111] Xu S. J. Heller E. A. (2019). Recent advances in neuroepigenetic editing. *Curr. Opin. Neurobiol.* 59 26–33. 10.1016/j.conb.2019.03.010 31015104 PMC12826455

[B112] Yamaguchi T. Sheen W. Morales M. (2007). Glutamatergic neurons are present in the rat ventral tegmental area. *Eur. J. Neurosci.* 25 106–118. 17241272 10.1111/j.1460-9568.2006.05263.x PMC3209508

[B113] Yim Y. Y. Teague C. D. Nestler E. J. (2020). *In vivo* locus-specific editing of the neuroepigenome. *Nat. Rev. Neurosci*. 21 471–484. 10.1038/s41583-020-0334-y 32704051 PMC7439525

[B114] You C. Vandegrift B. J. Zhang H. Lasek A. W. Pandey S. C. Brodie M. S. (2018). Histone deacetylase inhibitor suberanilohydroxamic acid treatment reverses hyposensitivity to gamma-aminobutyric acid in the ventral tegmental area during ethanol withdrawal. *Alcohol. Clin. Exp. Res.* 42 2160–2171. 10.1111/acer.13870 30103280 PMC6214766

[B115] Zhang T. A. Placzek A. N. Dani J. A. (2010). *In vitro* identification and electrophysiological characterization of dopamine neurons in the ventral tegmental area. *Neuropharmacology* 59 431–436. 10.1016/j.neuropharm.2010.06.004 20600174 PMC2946471

[B116] Zhang Y. (2003). Transcriptional regulation by histone ubiquitination and deubiquitination. *Genes Dev.* 17 2733–2740. 10.1101/gad.1156403 14630937

[B117] Zlotorynski E. (2019). Histone serotonylation boosts neuronal transcription. *Nat. Rev. Mol. Cell Biol.* 20:323. 10.1038/s41580-019-0124-4 30944456

